# Transcriptome Profiling of Pacu (*Piaractus mesopotamicus*) Challenged With Pathogenic *Aeromonas hydrophila*: Inference on Immune Gene Response

**DOI:** 10.3389/fgene.2020.00604

**Published:** 2020-06-09

**Authors:** Vito Antonio Mastrochirico-Filho, Milene Elissa Hata, Rafael Yutaka Kuradomi, Milena Vieira de Freitas, Raquel Belini Ariede, Daniel Guariz Pinheiro, Diego Robledo, Ross Houston, Diogo Teruo Hashimoto

**Affiliations:** ^1^Aquaculture Center, São Paulo State University (Unesp), Jaboticabal, Brazil; ^2^Faculty of Agricultural and Veterinary Sciences, São Paulo State University (Unesp), Jaboticabal, Brazil; ^3^The Royal (Dick) School of Veterinary Studies, The University of Edinburgh, Edinburgh, United Kingdom

**Keywords:** disease resistance, complement system, *de novo* assembly, RNA-Seq, aquaculture

## Abstract

Pacu (*Piaractus mesopotamicus*) is a Neotropical fish of major importance for South American aquaculture. Septicemia caused by *Aeromonas hydrophila* bacteria is currently considered a substantial threat for pacu aquaculture that have provoked infectious disease outbreaks with high economic losses. The understanding of molecular aspects on progress of *A. hydrophila* infection and pacu immune response is scarce, which have limited the development of genomic selection for resistance to this infection. The present study aimed to generate information on transcriptome of pacu in face of *A. hydrophila* infection, and compare the transcriptomic responses between two groups of time-series belonging to a disease resistance challenge, peak mortality (HM) and mortality plateau (PM) groups of individuals. Nine RNA sequencing (RNA-Seq) libraries were prepared from liver tissue of challenged individuals, generating ∼160 million 150 bp pair-end reads. After quality trimming/cleanup, these reads were assembled *de novo* generating 211,259 contigs. When the expression of genes from individuals of HM group were compared to individuals from control group, a total of 4,413 differentially expressed transcripts were found (2,000 upregulated and 2,413 downregulated candidate genes). Additionally, 433 transcripts were differentially expressed when individuals from MP group were compared with those in the control group (155 upregulated and 278 downregulated candidate genes). The resulting differentially expressed transcripts were clustered into the following functional categories: cytokines and signaling, epithelial protection, antigen processing and presentation, apoptosis, phagocytosis, complement system cascades and pattern recognition receptors. The proposed results revealing relevant differential gene expression on HM and PM groups which will contribute to a better understanding of the molecular defense mechanisms during *A. hydrophila* infection.

## Introduction

Disease outbreaks are considered a primary constraint and a continuous challenge to the growth of aquaculture sector (FAO, 2018). Detrimental effects due improper management practices, high-density culture systems and fluctuating water parameters have facilitated the spreading of bacteria or other pathogens, resulting in frequent and often severe disease outbreaks. This scenario has led to the billions of dollars in losses to aquaculture every year, that may exceed 40% of global capacity of production (Lafferty et al., 2015; Stentiford et al., 2017; Owens, 2019). While economic impact of disease outbreaks are under-reported for fish production in South America, a quarter of Brazilian large producers have reported moderate to high production losses due to disease outbreaks in the major aquaculture regions (Benavides and Maciel, 2015).

The incidence of sanitary problems linked to intensification of fish production from South America have resulted in large mortalities of Pacu (*Piaractus mesopotamicus*), one of the most important native fish species produced in Brazil (IBGE, 2016; Woynárovich and Van Anrooy, 2019). Pacu is considered a promising species for Brazilian aquaculture due to its large size, high quality meat and low-cost diet (Woynárovich and Van Anrooy, 2019). However, one of the main causes of disease associated with large mortalities for this species is the infection by *Aeromonas hydrophila* (Carraschi et al., 2012; Farias et al., 2016; Mastrochirico-Filho et al., 2019).

*Aeromonas hydrophila* is considered the etiological agent of Motile Aeromonas Septicemia (*MAS*), and is generally associated with symptoms such as reddened or rotten fins, small ulcerative lesions on the skin and hemorrhage, often followed by mortality of individuals within hours after manifestation of disease (Silva et al., 2012; Stratev and Odeyemi, 2017). Despite the lack of official data on the economic losses in fish production attributed to *A. hydrophila* infection, Brazilian fish farmers have reported that *MAS* prevalence is responsible for 20–30% of annual mortalities on pacu farming. However, insufficient knowledge on preventive measures and immune mechanisms of susceptibility and resistance are available to *MAS* (Belém-Costa and Cyrino, 2006; Farias et al., 2016; Zanuzzo et al., 2017; Mastrochirico-Filho et al., 2019).

RNA-Seq has been considered an effective technique for understanding the transcriptomic changes that occur during infection, exploring the activation of defense mechanisms involved in fast responses of infected hosts, even in organisms without a reference genome (Sudhagar et al., 2018). Over the last years, an increasing effort has been accomplished to characterize host-pathogen interaction by differentially expressed genes (DEGs) of fish species submitted to *A. hydrophila* resistance challenges, such as blunt snout bream *Megalobrama amblycephala* (Tran et al., 2015); grass carp *Ctenopharyngodon idella* (Dang et al., 2016); darkbarbel catfish *Pelteobagrus vachellii* (Qin et al., 2017); hybrid sturgeon (*Huso dauricus* × *Acipenser schrenckii*) (Jiang et al., 2018); and other species (Maekawa et al., 2019). These expression profiling have been considered useful to obtain information about the genomic makeup of immune responses at a molecular level, contributing to a better understanding of genetic resistance to disease, and guiding selective breeding strategies to produce more resistant strains. Additionally, in order to develop knowledge to suppress the disease, a better understanding of host-pathogen interaction between fish and bacteria, during critical periods of a disease outbreak is needed.

Therefore, the aim of the study was to characterize the transcriptome of pacu after *A. hydrophila* infection. A *de novo* assembly was necessary, since pacu is considered a non-model species and does not have a reference genome until the present moment. Further, the identification of differentially expressed genes involved in the response to this bacteria was performed aiming immune system genes, comparing differences between the actions of genomic mechanisms under two progressive time trends of a disease resistance challenge, peak mortality (HM) and mortality plateau (PM).

## Materials and Methods

### Ethics Statement

This study was conducted in strict accordance with the recommendations of the National Council for Control of Animal Experimentation (CONCEA) (Brazilian Ministry for Science, Technology and Innovation) and was approved by the Ethics Committee on Animal Use (CEUA number 19.005/17) of Faculdade de Ciências Agrárias e Veterinárias, UNESP, Campus Jaboticabal, SP, Brazil. All biosafety procedures related to the management of bacteria and infected animals were carried out. In addition, all measures related to the safety of the participants of the present study in handling the strains of bacteria were ensured.

### Generation of Pacu Families

Individuals used for RNA sequencing were obtained from an experimental challenge carried out in 14 full-sibling families of pacu, generated by a hierarchical mating scheme using 4 dams and 14 sires (3 or 4 sires per dam). Induced spawning was performed using carp pituitary extract dissolved in saline solution (0.9% NaCl) and applied in two dosages, with a 12 h interval (first and second dosage of 0.6 and 5.4 mg/kg, respectively). For sires, a single dosage was used, at the same time of the second dosage for dams, equivalent to 1.5 mg/kg of carp pituitary extract.

After hatching in 20 l conical fiberglass incubators, the larvae were fed with artemia nauplii for 20 days. Gradually, the feed was replaced by 50% of crude protein. In the fingerling stage, 1.2 mm pelleted feeds were used (40% of crude protein), being gradually replaced by 2 to 3 mm pelleted feeds (36% of crude protein) provided twice daily in 60 l tanks. Animals used in the experiment were pit-tagged to maintain the pedigree information during the challenge experiments. Laterally, fish were kept in 800 l fiberglass tanks at the Laboratory of Genetics in Aquaculture and Conservation (LaGeAC), at the São Paulo State University (UNESP), Jaboticabal city (São Paulo, Brazil).

### Experimental Challenge by *Aeromonas hydrophila*

Bacterial challenge was performed using a strain of *A. hydrophila* isolated from an outbreak of pacu aeromoniasis in a commercial facility from the São Paulo State, by the Laboratory of Microbiology and Parasitology of Aquatic Organisms, at UNESP, Jaboticabal city (São Paulo, Brazil). The strain of *A. hydrophila* was the same of that used by Mastrochirico-Filho et al. (2019). The strain was cultured in Agar Trypticase Soy (ATS), Vegitone (Sigma-Aldrich) for 24 h (28°C). The colony was then transferred to a nutrient tryptic soy broth (TSB) (Sigma-Aldrich) and cultured for 24 h (28°C). After bacteria growth, the culture was centrifuged at 5,000 × *g* for 10 min (4°C, in the Eppendorf Centrifuge 5810), forming a bacterial pellet suspended in a saline solution (PBS) and washed twice.

The challenge test performed in this study was carried out by intraperitoneal inoculation, according to protocols of Mastrochirico-Filho et al. (2019). The LD_50_ (lethal dose in 50% of individuals) was previously tested in 60 randomly chosen individuals from the same pacu families using concentrations adjusted by optical density of the solution at 0.400, 0.600, and 0.800 at 625 nm in spectrophotometer (2100 Unico, Japan). A sample of 100 μl of the LD50 was removed from the inoculum to perform serial dilutions and plate counts in duplicate on Trypticase Soy Agar (TSA).

Prior to challenge, the presence of *A. hydrophila* and other pathogens, such as *Flavobacterium columnare* and *Streptococcus agalactiae* in the populations was discarded checking subsamples of the populations by routine microbiological tests. In the experimental design of the challenge test, the individuals were distributed into three communal tanks of 2 m^3^ (length = 2 m, width = 1 m, depth = 1 m), where approximately ten individuals from each family were randomly distributed into each treatment tank. Prior to the inoculation of bacteria, fish were anesthetized with benzocaine (0.1 mg/l) and weighed. The mean weight of animals prior to the bacterial inoculation time was 23.0 ± 9.06 g. Individual fish were injected by intraperitoneal inoculation of the predefined LD_50_ dose of live cells of *A. hydrophila* (8 × 10^5^ CFU/g body weight), according to protocols carried out by Mastrochirico-Filho et al. (2019). Moreover, approximately ten fish from each family were also used as control and kept in a separated tank (called as control tank) of 2 m^3^ (length = 2 m, width = 1 m, depth = 1 m). Individuals of the control tank were injected by intraperitoneal inoculation of saline solution (PBS).

Each treatment and control tank were maintained with an independent water recirculation system, fitted with mechanical and biological filters, external aeration system, and controlled temperature using thermal controller connected to heaters (2 × 500 w). Water quality parameters were determined daily during 14 days of the challenge. Temperature, dissolved oxygen and pH were measured with a Multiparameter Water Quality Checker U-50 (Horiba, Kyoto, Japan). Water temperature was maintained at 30°C (*SD* = 0.5), with dissolved oxygen at 5.8 mg/L (*SD* = 1.0) and pH at 7.0 (*SD* = 1.1) during the challenge period. No water was exchanged during the challenge, but the tanks were topped off to compensate for evaporation. The control tank had the same condition when compared with treatment tanks, except for an antibacterial treatment through a UV filter.

Fish mortality was observed throughout the entire day (24 h) in the initial 3 days of challenge, and in intervals of 8 h in the remaining days of the challenge experiment. Fish that showed mortality by clinical signs of *A. hydrophila* infection (e.g., disequilibrium, hemorrhage, isolation from the group) were recorded and removed immediately from the tanks. Necropsy examination and microbiological tests were performed in a sub-sample of dead fish in order to confirm mortality by *A. hydrophila* and discard other pathogens. The isolation of bacteria was performed in a specific growth medium (phenol red agar and ampicillin) incubated at 28°C for 48 h. All surviving fish were examined externally for clinical signs of disease.

### Tissue Collection, RNA Isolation and Sequencing

According to experimental challenges by *A. hydrophila* in different fish species, the response of the immune system is very fast to this bacteria infection, occurring when mortality rates are generally detected in the first 24 h after exposure, whereas the mortality plateau generally occurs 72 h after infection (Mahapatra et al., 2008; Ødegård et al., 2010; Xiong et al., 2017; Mastrochirico-Filho et al., 2019). Therefore, fish presenting classical symptoms of *A. hydrophila* infection at 24 h after bacteria inoculation were collected and considered as belonging to HM (period of high mortality) group. When the mortality plateau occurred at 72 h after bacteria inoculation, fish were also collected and considered as belonging to PM (period of mortality plateau) group.

Laterally, three randomly selected families were sampled for RNA sequencing. Therefore, liver tissue from three individuals belonging to the HM group and three individuals belonging to the PM group were sampled from each family. Three control fish at each time trends of mortality were collected from the same families. Liver was collected from all individuals and stored in RNAlater^®^. This organ was chosen because it plays a critical role in the coordination of innate and adaptive immune responses (Kumar et al., 2017; Rebl and Goldammer, 2018), and it has been used in several studies involving transcriptome analysis in fish for a better understanding of the behavior of the immune system in face of bacterial infections (Tran et al., 2015; Maekawa et al., 2019; Jiang et al., 2020).

RNA was extracted using RNeasy Plus Universal Tissue Kit (Qiagen, Valencia, CA, United States) following the manufacturer’s specifications. After extraction, total RNA was treated with DNAse I to remove any DNA contamination. Total RNA was quantified by Qubit RNA Assay Kit in a Qubit 2.0 Fluorometer (Life Technologies, Carlsbad, CA, United States) and RNA integrity verified using a 2100 Bioanalyzer (Agilent Technologies). After mRNA extraction by poly-A tail selection, cDNA libraries were constructed using the TruSeq RNA Library Prep Kit protocol (Illumina). Six cDNA libraries were created from the combination of three pooled samples per family (family A, V, and R) diluted to the same concentration, for each time-series of mortality (HM and PM). At the same time, three cDNA libraries (one per family) were created for control samples, pooling three samples belonging to time-series (HM + PM), totalizing 9 cDNA libraries (Table 1). Sequencing was performed on the Illumina Hiseq^TM^ 2500 platform.

**TABLE 1:** Preparation of cDNA pooled libraries of *P. mesopotamicus* families (A, V, R) challenged on *A. hydrophila* infection.

Treatment	Treatment samples			Control samples		
	Family A	Family V	Family R	Family A	Family V	Family R
HM	3	3	3	3	3	3
PM	3	3	3	3	3	3
**Three pools per family:**						
Pool 1	HM treatment					
Pool 2	PM treatment					
Pool 3 (control)	HM + PM					

*The pools were constructed containing three individuals per family. Three cDNA libraries were constructed for control group (one per family) adding individuals belonging HM + PM time-trends of mortality.*

### Data Processing and Analysis

All RNA-Seq data generated for this study were deposited in the National Center for Biotechnology Information Sequence Read Archive under BioProject ID PRJNA632934. Bioinformatics pipelines are available in Supplementary Table S1. The quality of raw data was initially checked using FASTQC v. 0.11.9 (Andrews, 2010). Quality filtering and removal of residual Illumina adaptors were conducted on read pairs using TRIMMOMATIC v.0.36 (Bolger et al., 2014). Leading and trailing bases with a Phred score less than 20 were removed. Additionally, the read was trimmed if a sliding window average Phred score over four bases was less than 20. Following other trimming strategies adopted on studies approaching transcriptomic changes in fish submitted to disease infections, only pair reads greater than 36 bp were maintained (Robledo et al., 2014, 2018).

After filtering out low quality reads, the remained reads were used to assemble a *de novo* transcriptome using TRINITY v.2.9.1, adopting default parameters (Grabherr et al., 2011) as kmer-length value of 25 and contiguous sequences (contigs) longer than 200 bp. Read representation was evaluated by mapping the cleaned reads back to generated assembly with BOWTIE2 v.2.3.4.3 (Langmead and Salzberg, 2012). *A. hydrophila* genome (Yang et al., 2016), zebrafish ribosomal RNA (rRNA) sequences and pacu mitochondrial genome (da Pimentel et al., 2014) were then downloaded from the NCBI (National Center for Biotechnology Information) database and mapped against the assembly using BOWTIE2 v.2.3.4.3 (Langmead and Salzberg, 2012) to remove any contamination. Redundancy was reduced using CD-HIT-EST v.4.6.8 software by clustering sequences at 95% identity (Li and Godzik, 2006). Transcriptome statistics were generated using TRANSRATE v1.0.2 (Smith-Unna et al., 2016).

Annotation and homology searches of assembled transcripts were conducted using the TRINOTATE pipeline (Bryant et al., 2017). The assembled transcripts were translated into long open reading frames by TRANSDECODER (Haas et al., 2013). Homology searches were performed against the UniprotKB/Swiss-Prot database (BLASTX - E-value 1e^–3^) to identify known protein sequences (Altschul et al., 1990). Gene Ontology (GO) (Ashburner et al., 2000), eggNOG/COG (Non-supervised orthologous groups) (Powell et al., 2012), and Kyoto Encyclopedia of Genes and Genomes (KEGG) (Kanehisa et al., 2012) databases were used to assign functions and biology pathways to transcripts.

KALLISTO v0.43.1 software was used to compute expression levels for each pooled samples (HM, PM and control) by pseudoalignments (Bray et al., 2016). Initially, an index was built with default k-mer size based on the contigs assembled by TRINITY. After, in order to quantify abundances of transcripts, TPM (transcripts per million reads) expression levels of genes for each of the nine pooled samples were computed. DESeq2 (Love et al., 2014) used negative binomial distribution, data normalization and FDR (false recovery rate) correction through Benjamini-Hochberg (BH) method, to identify genes differentially expressed comparing the gene expression from pooled samples belonging to HM and PM groups against pooled samples from control groups. To control false discovery rates of multiple-hypothesis testing, the *p*-values calculated by DESeq2 were processes to *q*-values. Then, the list of differentially expressed transcripts were generated with cutoff *q*-values less than 0.05 and log_2_FC (fold change (condition/control) for a gene) > 1 or log_2_FC < −1) as described in similar transcriptome studies (Marancik et al., 2015; Polinski et al., 2016; Jiang et al., 2020).

## Results

### Disease Challenge Experiment

During the *A. hydrophila* resistance challenge, moribund individuals demonstrated lethargic behavior, erratic swimming and red spotted skin lesions on the operculum, head, fins and gills. No symptoms related to infection were reported in individuals in the unchallenged control group. High mortality rates (93.1%) were observed mainly at the first day after inoculation (HM period). Resistance rates of sampled families for RNA sequencing ranged from 16 to 32%. *A. hydrophila* was successfully isolated from infected individuals, confirming the bacterial infection. The last mortality was recorded at approximately 65 h post inoculation (PM period).

### Sequencing and *de novo* Assembly

RNA sequencing, generated from liver samples, yielded between 15 and 20 million paired-end reads per pooled library. Approximately 12% of low-quality reads (quality score < 20) were removed. This resulted in approximately 143 million (87.8%) high quality paired-end reads, with a total length of 39.81 × 10^9^ bp to be explored in the analysis of the pacu transcriptome (Table 2). *De novo* assembly produced 238,054 contigs longer than 200 bp, totalizing 169,652,373 assembled bases. Additionally, the average contig length was 712.6 bp with an N50 value of 1,296 bp. Approximately 92% of the filtered reads mapped back to the assembly, of which 76% mapped adequately (both pair reads aligned in the correct orientation and in the same contig) (Table 3).

**TABLE 2:** RNA-seq statistics in *P. mesopotamicus* families (A, V, R) challenged on *A. hydrophila* infection.

Library	Condition	Raw reads	Filtered reads (%)
C-A	Control	16,636,300	14,741,119 (88.6)
C-V	Control	18,560,164	16,282,254 (87.7)
C-R	Control	17,224,277	15,222,441 (88.4)
HM-A	Peak mortality	20,012,222	17,463,031 (87.3)
HM-V	Peak mortality	19,385,555	17,086,377 (88.1)
HM-R	Peak mortality	15.571.531	13,619,104 (87.5)
PM-A	Mortality plateau	16,760,301	14,861,247 (88.7)
PM-V	Mortality plateau	19,393,490	17,046,959 (87.9)
PM-R	Mortality plateau	19,998,031	17,244,459 (86.2)
Total		163.541.871	143.566.991 (87.8)

**TABLE 3:** Summary of *de novo* assembly statistics of *P. mesopotamicus* liver transcriptome in *A. hydrophila* disease challenge.

Features	Results
Total bases of filtered reads	39,806,523,666
Total assembled bases	169,652,373
Number of transcripts	238,054
Average transcript length	713
Maximum transcript length	31,450
N50	1296
% reads mapped to the assembly	∼92
% paired-reads adequately mapped	76

Posteriorly, 115 sequences mapped to the *A. hydrophila* genome, 20 sequences mapped to pacu mitochondrial genome and 90 transcripts identified as part of zebrafish ribosomal RNA (rRNA) were identified as contaminants, and therefore were excluded of the analysis. In addition, CD-HIT-EST clustered around 11.2% (26,570) of the transcripts resulting in a non-redundant assembly containing 211,259 sequences.

### Gene Annotation

**FIGURE 1:**
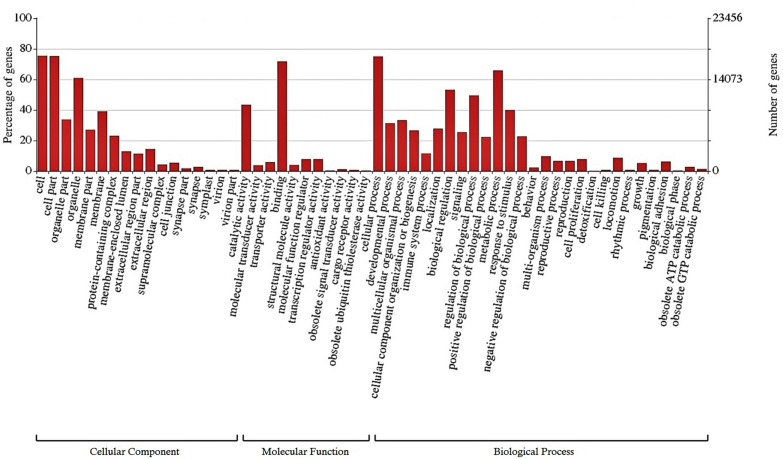
Functional classification of the liver transcriptome of pacu (*P. mesopotamicus*) exposed to *A. hydrophila* infection. The transcripts were classified in the *x*-axis, according to Gene Ontology (GO) categories at level 2: cellular component, molecular function and biological process (E-value < 1e-3). The primary *y*-axis represents the percentage of genes classified in each subcategory, whereas the secondary *y*-axis represents the number of classified genes.

A total of 25,807 (12.2%) transcripts were annotated based on currently known proteins in the UniprotKB/Swiss-Prot database (Supplementary Table S2). Most of the annotate transcripts (15,547; 60%) showed significant homology with *Astyanax mexicanus* proteins, followed by *Danio rerio*, *Cyprinus carpio*, *Clupea harengus*, and *Salmo salar* proteins with 2,340 (9%); 1,368 (5.3%); 574 (2.2%); and 422 (1,6%) matched transcripts, respectively. Laterally, 23,420 transcripts were assigned into one or more GO terms classifying 20,851 transcripts into biological processes, 20,604 transcripts into molecular function and 19,884 transcripts into cellular component category (Figure 1). The most frequent GO term subcategories were cellular process, metabolic process and biological regulation for biological process; binding and catalytic activity for molecular function; and cell, cell part and organelle for cellular component. Furthermore, GO annotation revealed pertinent information on 2,726 transcripts assigned to immune system processes.

**FIGURE 2:**
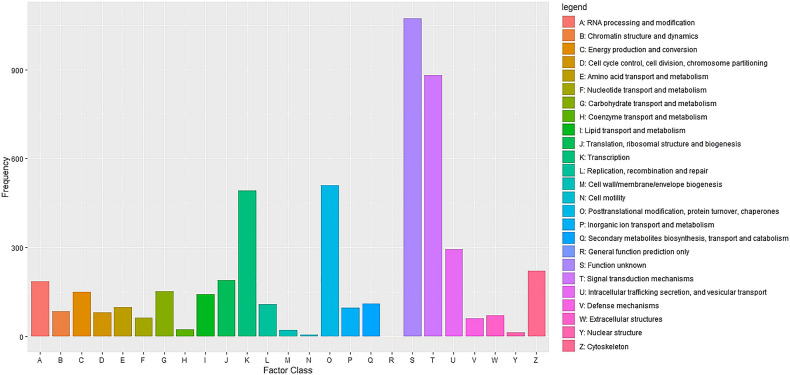
Functional classification of the pacu liver transcriptome exposed to *A. hydrophila* infection based on eggnog/COG orthologous groups (E-value < 1e-3). 4,931 pacu transcripts were classified in 25 functional categories arranged in the *x*-axis. The *y*-axis presents the frequency of each represented category.

**FIGURE 3:**
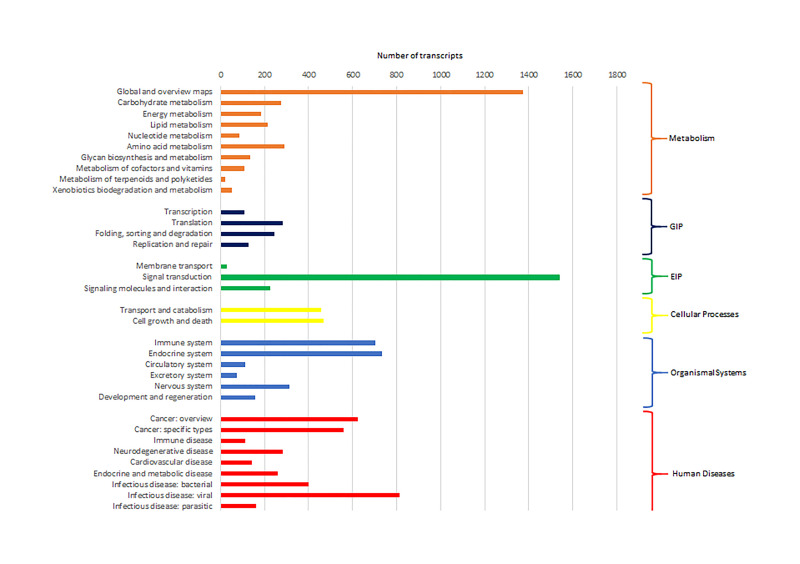
Number of pacu transcripts (*x*-axis) assigned to each KEGG pathway arranged in the *y*-axis. The categories GIP and EIP stand for “Genetic Information Processing” and “Environmental Information Processing.”

Cluster of Orthologous Groups (COG) using the EggNOG database assigned 4,931 transcripts to 25 COG functional categories (Supplementary Table S3 and Figure 2). The largest clusters were composed by transcripts of unknown function (1,075 transcripts, 21.8%), followed by clusters related to signal transduction mechanisms (884 transcripts, 17.9%), post-translational modification and protein turnover (chaperones systems) (510 transcripts, 10.3%). Other clusters related to transcripts involved in intracellular trafficking secretion and vesicular transport (296 transcripts, 6.0%), RNA processing and modification (187 transcripts, 3.8%) and defense mechanisms (61 transcripts, 1.2%) were well represented. Further, 8,422 transcripts were mapped to 405 KEGG pathways (Supplementary Table S4). The most represented KEGG pathways are shown in Figure 3. “Signal Transduction” pathway had the largest number of matched transcripts (1,540, 18.3%), followed by “Global and Overview Maps” (1,374, 16.3%), “Infectious Disease (viral)” (814, 9.6%), “Endocrine System” (732, 8.7%), and “Immune System” (703, 8.3%) pathways.

### Differential Gene Expression Analysis

**FIGURE 4:**
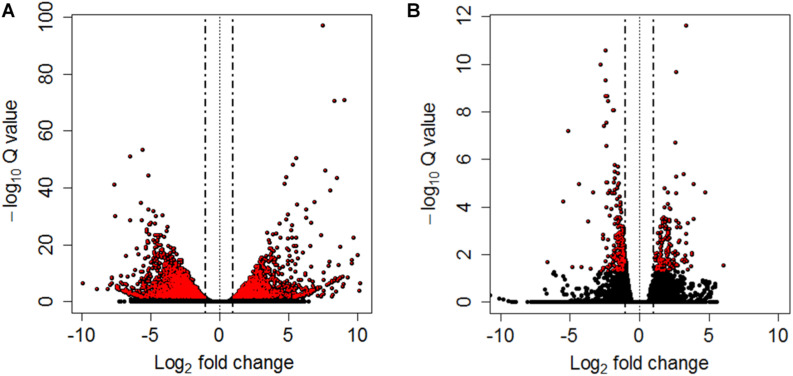
Volcano plots between controls and HM **(A)** and PM **(B)** pacu (*P. mesopotamicus*) challenged with *A. hydrophila*. Each dot represents the expression (*x*-axis, Log_2_ fold change), and significance (*y*-axis, −Log_10_ *Q*-value) of each transcript. Differentially expressed transcripts (*q*-value < 0.05) are red.

Differential expression analyses between pooled samples belonging to control and HM group showed 4,413 differentially expressed transcripts, where 2,000 transcripts were up-regulated (45.3%) and 2,413 transcripts were down-regulated (54.7%) in HM group (*q*-value < 0.05, log_2_FC > | 1|) (Figure 4A, Table 4, and Supplementary Table S5). Additionally, 433 transcripts were differentially expressed between PM group and control fish, with 155 up-regulated (35.8%) and 278 down-regulated (64.2%) transcripts considering the PM group (*q*-value < 0.05, log_2_FC > |1|) (Figure 4B, Table 4, and Supplementary Table S6).

**TABLE 4:** Number of differentially expressed transcripts of pacu (*P. mesopotamicus*) after infection with *A. hydrophila*.

Genes differentially expressed	Total	Upregulated	Downregulated
HM	4,413	2,000	2,413
PM	433	155	278
Only in HM fish	4,271	1,959	2,312
Only in PM fish	291	104	187

*Comparisons of HM and PM fish vs. control. Upregulations means more expression in the HM/PM animals when compared to controls (transcript q-value < 0.05). LFC > 1 were considered upregulated genes and LFC < −1 were considered downregulated genes.*

A significant fraction of the differentially expressed transcripts showed log_2_FC > | 5|, specifically 400 genes (9%) differentially expressed for HM group, and 5 genes (1.2%) differentially expressed in PM group. Further, a total of 4,271 genes (96.8%) were differentially expressed only in HM group, whereas 291 genes (67.2%) were differentially expressed exclusively in PM group (Figures 4A,B and Table 4).

In this study, only genes specifically related to the immune responses against *A. hydrophila* and differentially expressed in HM (Table 5) and PM (Table 6) groups were considered. Therefore, 57 transcripts identified as immune transcripts were considered differentially expressed in the HM group (31 up-regulated and 26 down-regulated transcripts) (*q*-value < 0.09) (Table 5), whereas 23 immune transcripts were considered differentially expressed in the PM group (15 up-regulated and 8 down-regulated transcripts) (*q*-value < 0.07) (Table 6). For didactic purposes, these transcripts were grouped and classified according to the important immune mechanisms that may be related to fighting infections such as those caused by *A. hydrophila*, such as: cytokines and signaling, epithelial protection, antigen processing and presentation, apoptosis, phagocytosis, complement system cascades and pattern recognition receptors (Tables 5, 6).

**TABLE 5:** List of the most significant differentially expressed immune system transcripts in HM group of *P. mesopotamicus* exposed to *A. hydrophila* infection vs. the controls.

Category	Gene name	Log_2_ Fold	Diff	*Q*-value
Epithelial protection	Guanylin	10.11	UP	0.0002
	NADPH oxidase organizer 1	9.29	UP	3.33e-09
	Protein-glutamine gamma-glutamyltransferase K	5.27	UP	2.49e-23
	Microfibril-associated glycoprotein 4	3.18	UP	2.61e-10
	Transmembrane serine protease 4	–5.37	DOWN	4.72e-20
Cytokines and signaling	Interleukin-1 receptor type 2	7.86	UP	5.02e-07
	Prostaglandin E synthase	7.45	UP	6.25e-98
	Relaxin-3	6.85	UP	7.69e-36
	C-C motif chemokine 20	6.50	UP	1.78e-08
	C-C motif chemokine 24	4.63	UP	1.78e-06
	Interleukin-1 beta	4.39	UP	0.001
	C-C motif chemokine 13	4.19	UP	0.004
	Interleukin-1 receptor type 1	3.56	UP	1.82e-06
	C-X-C motif chemokine 10	3.36	UP	0.01
	Carcinoembryonic antigen-related cell adhesion molecule 20	2.50	UP	1.87e-09
	C-X-C motif chemokine 14	–4.26	DOWN	6.85e-19
	Cytokine-dependent hematopoietic cell linker	–4.21	DOWN	0.08
	C-C chemokine receptor type 5	–3.49	DOWN	0.001
	Chemokine-like receptor 1	–3.46	DOWN	0.06
	C-X-C motif chemokine 11	–3.17	DOWN	0.06
	Atypical chemokine receptor 4	–2.70	DOWN	0.03
Apoptosis	Lactate dehydrogenase A	5.58	UP	7.92e-35
	Protein KIBRA	3.37	UP	8.96e-17
	Apoptosis-associated speck-like protein containing a CARD	2.73	UP	3.20e-06
	Perforin-1	–4.95	DOWN	0.0004
Phagocytosis	Monocarboxylate transporter 4	6.57	UP	2.13e-20
	C-type lectin domain family 4 member M	3.76	UP	1.50e-15
	Cytochrome B-245 beta chain	3.64	UP	1.73e-21
	Carnitine O-acetyltransferase	–4.38	DOWN	1.65e-20
	Macrophage migration inhibitory factor	–2.30	DOWN	6.23e-05
Pattern recognition receptor	Interleukin 1 receptor associated kinase 3	4.40	UP	2.31e-23
	Interleukin-1 receptor-associated kinase 1	2.72	UP	0.02
	Toll-interacting protein	1.75	UP	6.50e-05
	Protein NLRC3	–3.21	DOWN	0.02
Complement system	Tissue factor pathway inhibitor 2	3.93	UP	1.87e-18
	Complement component C6	2.70	UP	1.87e-06
	Coagulation factor XI	–4.99	DOWN	2.21e-28
	Complement C1q subcomponent subunit B	–4.04	DOWN	0.0001
	Coagulation factor VII	–4.00	DOWN	1.35e-17
	Complement C1q subcomponent subunit A	–3.65	DOWN	0.06
	Coagulation Factor IX	–3.53	DOWN	7.52e-20
	Complement C1r-A subcomponent	–2.49	DOWN	0.02
	Complement C3	–1.78	DOWN	4.48e-05
	Complement factor I	–1.64	DOWN	0.002
	Complement C4	–1.36	DOWN	0.005
	Complement C4-B	–1.35	DOWN	0.003
Antigen processing and presentation	Polyhomeotic-like protein 2	7.63	UP	5.51e-47
	Heat shock 70 kDa protein	6.26	UP	2.60e-30
	S-adenosylmethionine synthase 1	5.16	UP	7.61e-26
	Purine nucleoside phosphorylase	4.82	UP	1.05e-29
	Adhesion G protein-coupled receptor E1	4.15	UP	0.0006
	CD97 antigen	2.28	UP	1.30e-05
	Cathepsin-O	–5.19	DOWN	3.74e-45
	Retinol-binding protein 4	–4.12	DOWN	6.11e-06
	MHC class I-related gene protein	–3.28	DOWN	0.01
	CD48 antigen	–3.12	DOWN	1.77e-09
	B-cell linker protein	–2.11	DOWN	0.03

**TABLE 6:** List of most significant differentially expressed immune system transcripts in PM group of *P. mesopotamicus* exposed to *A. hydrophila* infection vs. the controls.

	Category and Gene name	Log_2_ Fold	Diff	*Q*-value
Cytokines and signaling	TAB2 - TGF-beta-activated kinase 1 and MAP3K7-binding protein 2	1.91	UP	0.0007
	Innate immunity activator protein	2.60	UP	0.01
	Suppressor of cytokine signaling	–2.38	DOWN	0.0001
Apoptosis	E3 ubiquitin-protein ligase	2.69	UP	0.0007
	Ankyrin repeat and KH domain-containing protein 1	1.46	UP	0.06
	Krueppel-like factor 15	–2.13	DOWN	0.0002
	Glyceraldehyde 3-phosphate dehydrogenase	–1.43	DOWN	0.0003
Phagocytosis	Rho GTPase-activating protein 35	3.16	UP	4.15e-06
	Natural resistance-associated macrophage protein 2	2.00	UP	2.47e-05
	25-hydroxyvitamin D-1 alpha hydroxylase	–5.12	DOWN	6.34e-08
	F173B - Protein FAM173B	–2.00	DOWN	0.0001
	Macrophage migration inhibitory factor	–1.58	DOWN	0.001
Pattern recognition receptor	Ladderlectin	5.97	UP	0
	DENN domain-containing protein 5B	1.91	UP	0.0003
	Proto-oncogene c-Fos	1.68	UP	0.0004
Complement system	Coagulation factor XI	1.62	UP	0.0003
	Plasma kallikrein	1.82	UP	1.66e-05
Antigen processing and presentation	Early growth response protein 1	3.35	UP	2.30e-12
	Rho GTPase-activating protein 5	2.14	UP	0.0003
	B-cell lymphoma 6 protein homolog	2.02	UP	0.02
	High affinity immunoglobulin gamma Fc receptor I	1.33	UP	0.04
	Hemopexin	–2.58	DOWN	3.92e-08
	Adenosylhomocysteinase B	–1.51	DOWN	1.00e-05

## Discussion

*De novo* assembly from pacu liver RNA sequencing generated a high-quality transcriptome containing 211,259 transcripts, presenting an N50 value of 1,296 bp and average length of 713 bp. Moreover, default settings (kmer-length fixed to 25 and minimum contig length of 200 bp) used by the Trinity software provided an optimal assembly, as they subsequently allowed 92% of the reads to be mapped back to the transcriptome, having few transcriptomic information missing. The evident quality of the transcriptome was similar to other RNA-seq studies involving infections in fish (Wang et al., 2013; Kumar et al., 2017; Song et al., 2017). Therefore, this immune-enriched *de novo* pacu liver transcriptome can be used as framework to investigate transcriptomic changes upon infection by *A. hydrophila* and other pathogens.

This is the first study on gene expression of pacu infected by *A. hydrophila*. The choice to focus only on genes related to the immune system was due to the generation of subsidies for future studies on application of immunostimulating treatments that could increase the pacu resistance when exposed to infectious outbreaks of *A. hydrophila.* Additionally, this results will contribute to develop effective strategies to protect pacu production from this threatening bacterial disease, and could be exploited through future development of breeding programs to increase the genetic resistance of pacu to *A. hydrophila* infection. Our results were similar to previous transcriptomic analyses in different species of fish infected by *A. hydrophila* (Tran et al., 2015; Dang et al., 2016; Qin et al., 2017; Jiang et al., 2018; Maekawa et al., 2019) showing transcripts related to innate immune system, which consist in a complex set of pathways that mediate the activation of phagocytosis, inflammation and destruction of the pathogen by antimicrobial substances, stimulating adaptive immune responses (Tort et al., 2003). In order to facilitate the discussion we have grouped these differentially expressed genes in categories based on their putative molecular mechanisms.

### Cytokines and Signaling

Cytokines are small proteins released by immune cells acting as signaling molecules that regulate immunity, inflammation and hematopoiesis. We found the *Interleukin-1 receptor type 1* (*IL1R1*) and *Interleukin-1 receptor type 2* (*IL1R2*) transcripts up-regulated in HM and PM groups. These transcripts are part of the Interleukin-1 receptor complex, involved in many cytokine-induced inflammatory responses.

We also found four chemokines upregulated and four downregulated in HM group. Chemokines are cytokines that regulate the leukocyte migration and the differentiation of recruited cells under inflammatory conditions, in order to orchestrate the first steps of both innate and adaptive immune responses (Bird and Tafalla, 2015). Further, we found downregulated genes involved in chemokine activation in HM group, and one transcript related to the negative regulation of cytokines (*Suppressor of cytokine signaling, SOCS1*), downregulated in PM group. Excess production of anti-inflammatory cytokines can compromise the ability to clear microorganisms through suppression of immune cell function. If a balance is not maintained, the result is either an excessive proinflammatory response or an immunosuppression (Chaudhry et al., 2015).

### Phagocytosis

Phagocytosis is an essential cellular process that captures and destroys foreign particles, such as small unicellular organisms. One of the major effects of *A. hydrophila* infection in fish is characterized by the production of toxins that can cause severe tissue destruction, affecting the epithelial cells due to the immune phagocytic activity in response to the bacteria (Cipriano et al., 2001; Abdelhamed et al., 2017). According to the results, *Natural resistance-associated macrophage protein 2* (*NRAMP2*) transcript was upregulated only in PM group. This protein has been previously considered an important regulator of macrophages in the response to *A. hydrophila* (Jiang et al., 2018). Further, a previous study on *cytochrome B-245 beta chain* (*CYBB*) transcription, upregulated in HM group, revealed that deficiency in the production of the protein impairs phagocytosis-mediated defense against *A. hydrophila*. This means that even if the phagocytes are able to phagocytize the bacteria, *A. hydrophila* can survive into the phagocytic vacuoles (Jiang et al., 2018).

### Pattern Recognition Receptors

*Interleukin-1 receptor-associated kinase 1* (*IRAK1*) transcript is directly involved in the recognition of pathogen-derived products and the consequent initiation of immune and inflammatory responses. This protein is considered part of Toll-like receptor complex, and it is usually expressed on sentinel cells, such as macrophages and dendritic cells, which recognize structurally conserved molecules derived from pathogens. *Interleukin 1 receptor-associated kinase 3* (*IRAK3*), which inhibits the dissociation of *IRAK1* from the Toll-like receptor signaling complex (Shan et al., 2015), is upregulated in HM group. Therefore, these results can suggest that these genes are involved in the induction of the inflammatory response in response to *A. hydrophila* infection in pacu.

### Complement System

The complement system is a key component of the innate immune system of fish. The activation of the complement pathway leads to opsonization of pathogens, recruitment of inflammatory and immune competent cells, and direct killing of pathogens through lysis (Merle et al., 2015). In the present study, practically all complement transcripts identified were downregulated in HM group (especially C1, C3, and C4), suggesting that complement pathways is not activated following *Aeromonas hydrophila* infection, which is different than reported in previous studies considering other fish species (Tran et al., 2015; Dang et al., 2016; Jiang et al., 2018). Complement system inactivation in HM group could be an important factor leading to the failure of the defense system, and consequent host death during the PM time-series that we found in the present study. However, further studies need to be performed in order to validate these genes and corroborate this hypothesis. In the same way, several coagulation factors were also downregulated in HM group, such as *Coagulation factor VII* (*F7*), *Coagulation Factor IX* (*F9*), and *Coagulation Factor XI* (*F11*), indicating the absence of blood coagulation, and favoring the occurrence of hemorrhages, as observed in HM group with individuals presenting clinical signs of *A. hydrophila* infection.

## Conclusion

Transcriptome analysis by RNA-seq allowed the identification and comparative quantification of gene expression in pacu infected by *A. hydrophila*. *De novo* liver transcriptome assembled in this study enables capturing expression levels of specific transcripts associated with important immune pathways, such as the coagulation factor and complement system cascades. Using this transcriptome, we have described for the first time the response of pacu infected by *A. hydrophila* in two critical periods of infection (PM and HM) comparing individuals belonging two distinct groups based on different phases of bacterial infection (acute mortality and plateau of mortality). To our knowledge this is the first descriptive study on transcriptome analysis of infected pacu that may serve as a reference to understand the genetic mechanisms underlying the host-pathogen interaction. The differences in the transcriptomic responses to a standardized *A. hydrophila* challenge was able to provide a initial data set to begin to understand the impact of selective breeding on genetic basis of disease resistance. Therefore, further studies trying to connect the genomic and genetic basis of resistance to *A. hydrophila* are fundamental to enable the implementation of *A. hydrophila* resistance into pacu breeding programs. Despite these limitations, these results may suggest possible suppression of genes belonging to the immune system that may contribute to susceptibility to infection, as for example genes related with complement system and coagulation factor cascades. Such supression of important immune system genes will serve as subsidies for future studies aiming at the use of prophylactic and immunostimulating treatments that could increase the pacu resistance when exposed to infectious outbreaks of *A. hydrophila*.

## Data Availability Statement

All RNA-Seq data generated for this study were deposited in the National Center for Biotechnology Information Sequence Read Archive under BioProject ID PRJNA632934. Bioinformatics pipelines are available in Supplementary Table S1.

## Ethics Statement

This study was conducted in strict accordance with the recommendations of the National Council for Control of Animal Experimentation (CONCEA) (Brazilian Ministry for Science, Technology and Innovation) and was approved by the Ethics Committee on Animal Use (CEUA number 19.005/17) of Faculdade de Ciências Agrárias e Veterinárias, UNESP, Campus Jaboticabal, SP, Brazil.

## Author Contributions

VM-F and DH: conceptualization, funding acquisition, and roles/writing – original draft. VM-F, DR, RH, and DH: formal analysis. VM-F, MH, RK, MF, RA, and DP: investigation and methodology. DH: project administration. DH, DR, and RH: supervision. All authors: writing – review and editing.

## Conflict of Interest

The authors declare that the research was conducted in the absence of any commercial or financial relationships that could be construed as a potential conflict of interest.
